# Prognostic impact of severe neutropenia in colorectal cancer patients treated with TAS-102 and bevacizumab, addressing immortal-time bias

**DOI:** 10.1186/s12885-023-11618-3

**Published:** 2023-11-08

**Authors:** Daichi Watanabe, Hironori Fujii, Koichi Ohata, Hirotoshi Iihara, Akitaka Makiyama, Ryo Kobayashi, Chiemi Hirose, Shiori Hishida, Serika Matsuoka, Jesse Yu Tajima, Shigeru Kiyama, Takao Takahashi, Akio Suzuki, Nobuhisa Matsuhashi

**Affiliations:** 1 Department of Pharmacy, Gifu University Hospital, Yanagido 1-1, Gifu, 501-1194 Japan; 2 Cancer Center, Gifu University Hospital, Gifu, Japan; 3 Laboratory of Advanced Medical Pharmacy, Gifu Pharmaceutical University, Gifu, Japan; 4 Department of Gastroenterological Surgery/Pediatric Surgery, Gifu University Graduate School of Medicine, Gifu, Japan; 5 Department of Surgery, Ibi Kousei Hospital, Gifu-Seino Medical Center, Gifu, Japan

**Keywords:** Colorectal neoplasms, Trifluridine tipiracil drug combination, Bevacizumab, Neutropenia, Drug-related side effects and adverse reactions, Immortal-time bias

## Abstract

**Background:**

Several studies have reported an association between severe neutropenia and long-term survival in patients treated with trifluridine-tipiracil (TAS-102). Because some of these studies failed to address immortality time bias, however, their findings should be interpreted with caution. Additionally, the association between severe neutropenia and survival in patients receiving TAS-102 in combination with bevacizumab (Bmab) remains unclear.

**Patients and methods:**

We conducted a single-center retrospective cohort study in patients with colorectal cancer who received Bmab + TAS-102. We compared overall survival (OS) between patients who developed grade ≥ 3 neutropenia during the treatment period and those who did not. To account for immortal time bias, we used two approaches, time-varying Cox regression and landmark analysis.

**Results:**

Median OS was 15.3 months [95% CI: 14.1–NA] in patients with grade ≥ 3 neutropenia and 10.0 months [95% CI: 8.1–NA] in those without. In time-varying Cox regression, onset grade ≥ 3 neutropenia was significantly related to longer survival after adjustment for age and modified Glasgow Prognostic Score. Additionally, 30-, 60-, 90-, and 120-day landmark analysis showed that grade ≥ 3 neutropenia was associated with longer survival after adjustment for age and modified Glasgow Prognostic Score, with respective HRs of 0.30 [0.10–0.90], 0.65 [0.30–1.42], 0.39 [0.17–0.90], and 0.41 [0.18–0.95].

**Conclusion:**

We identified an association between long-term survival and the development of severe neutropenia during the early cycle of Bmab + TAS-102 using an approach that addressed immortality time bias.

**Supplementary Information:**

The online version contains supplementary material available at 10.1186/s12885-023-11618-3.

## Implications for practice

Our study found that the occurrence of grade ≥ 3 neutropenia during the treatment period of Bmab + TAS-102 was significantly associated with long-term survival in patients with colorectal cancer. For patients who do not develop neutropenia during the early cycle of Bmab + TAS-102, increasing TAS-102 dosage to improve treatment outcomes may warrant consideration.

## Introduction

Trifluridine-tipiracil (TAS-102) is an oral antitumor agent composed of the thymidine-based nucleic acid analog trifluridine and the thymidine phosphorylase inhibitor tipiracil hydrochloride in a molar ratio of 1:0.5. In Phase III clinical trials, TAS-102 was clinically superior to placebo in overall survival (OS) in patients with metastatic colorectal cancer refractory to standard therapies, including fluoropyrimidines, irinotecan, and oxaliplatin [1, 2].

Our previous retrospective study compared TAS-102 in combination with bevacizumab (Bmab + TAS-102) and TAS-102 monotherapy using a propensity-matched cohort. Results indicated that Bmab + TAS-102 significantly improved OS in patients with metastatic colorectal cancer refractory to standard therapies (hazard ratio [HR], 0.24 [95% CI 0.12–0.52]; *p* < 0.001) [3]. Additionally, a randomized phase 2 study demonstrated that Bmab + TAS-102 prolonged progression-free survival (PFS) (HR 0.45 [95% CI 0.29–0.72]; *p* = 0.0015) compared with TAS-102 monotherapy [4].

The most common adverse event associated with TAS-102 monotherapy is neutropenia, with 37.9% of patients treated with TAS-102 monotherapy experiencing grade ≥ 3 neutropenia in the RECOURSE trial [1]. Moreover, the incidence of grade ≥ 3 neutropenia tended to be higher on combination of Bmab with TAS-102 compared with TAS-102 monotherapy (67% vs. 38%) [4].

Several retrospective cohort studies reported an association between the occurrence of neutropenia during TAS-102 monotherapy or Bmab + TAS-102 therapy and long-term survival [5–8]. These findings should be interpreted with caution, however, because classification of the group without neutropenia to the group with neutropenia group after treatment initiation may cause a problem known as immortal time bias [9].

In this study, we aimed to determine the association between the onset of severe neutropenia and survival in patients with colorectal cancer who received Bmab + TAS-102 using an approach that addresses immortal time bias.

## Patients and methods

### Study design

The study was conducted under a retrospective cohort design at a single center in patients diagnosed with metastatic colorectal cancer who were refractory to standard chemotherapy regimens and received Bmab + TAS-102. Survival duration was compared between patients with and without grade ≥ 3 neutropenia during the treatment period, with the onset of neutropenia considered as a time-dependent covariate.

### Setting and participants

The data were obtained from electronic medical records at Gifu University Hospital. The study population consisted of patients with colorectal cancer who were refractory to standard therapies, including fluoropyrimidine, irinotecan, oxaliplatin, anti-VEGF therapy, and anti-EGFR therapy (in cases of tumors with wild-type RAS), and who received Bmab + TAS-102 between March 2016 and June 2021. The exclusion criterion for this study was a reduction in the initial dose of TAS-102.

### Variables

The primary endpoint of the study was OS, defined as the time elapsed from the initiation of Bmab + TAS-102 to death. PFS was defined as the time from the initiation of Bmab + TAS-102 to the first date of radiological or clinical progression or time of death. For OS and PFS, patients who were lost to follow-up or survived until the end of the observation period were censored. Tumor response was assessed according to the guidelines outlined in Response Evaluation Criteria in Solid Tumors version 1.1 [10]. Disease control rate was defined as the proportion of patients with a complete or partial response, or stable disease. Neutropenia was graded according to the Common Terminology Criteria for Adverse Events version 5.0 [11], with grade ≥ 3 neutropenia defined as severe neutropenia. Potential confounding factors, such as age and modified Glasgow Prognostic Score (mGPS), were considered in the analysis. The latter is a widely recognized prognostic predictor of colorectal cancer which is calculated based on serum albumin and CRP levels: patients with a serum albumin level greater than 3.5 g/dL and CRP less than 1.0 mg/dL were classified as mGPS 0, those with either decreased albumin levels or increased CRP levels as mGPS 1, and those with both decreased albumin levels and elevated CRP levels as mGPS 2 [12].

### Statistical methods

In the present study, the exposure group consisted of patients who experienced grade ≥ 3 neutropenia during the follow-up period. However, this raises concerns of immortal time bias [13]. To address this potential, our primary analysis utilized a time-varying Cox regression model rather than assuming that hazard ratios remained unchanged over time [13, 14]. The Simon and Makuch method [14] was employed to graphically depict survival curves for time-dependent covariates. This modified Kaplan–Meier survival curve is a more accurate representation of the difference in survival curves than simply assuming a fixed covariate and plotting Kaplan–Meier curves [15].

Additionally, landmark analysis was performed as sensitivity analysis [16, 17]. In our study, landmark time points were established at 30, 60, 90, and 120 days post-treatment initiation. Outcome events after each landmark were included in the analysis. Patients who developed grade ≥ 3 neutropenia prior to each landmark were classified into the neutropenia group, while those who did not develop grade ≥ 3 neutropenia prior to each landmark were classified into the non-neutropenia group. Hazard ratios for death between the two groups were calculated using Cox proportional hazards regression analysis.

As no specific hypothesis was predetermined regarding the anticipated effect in this study, no sample size calculation was performed. Data analysis was conducted using R software version 4.2.2. In all analyses, a two­tailed *p* value of less than 0.05 was deemed to be significant. Since the statistical analysis plan did not include a provision for correcting multiple comparisons for secondary outcomes, these results cannot be conclusively interpreted.

## Results

### Patients

**Fig. 1:**
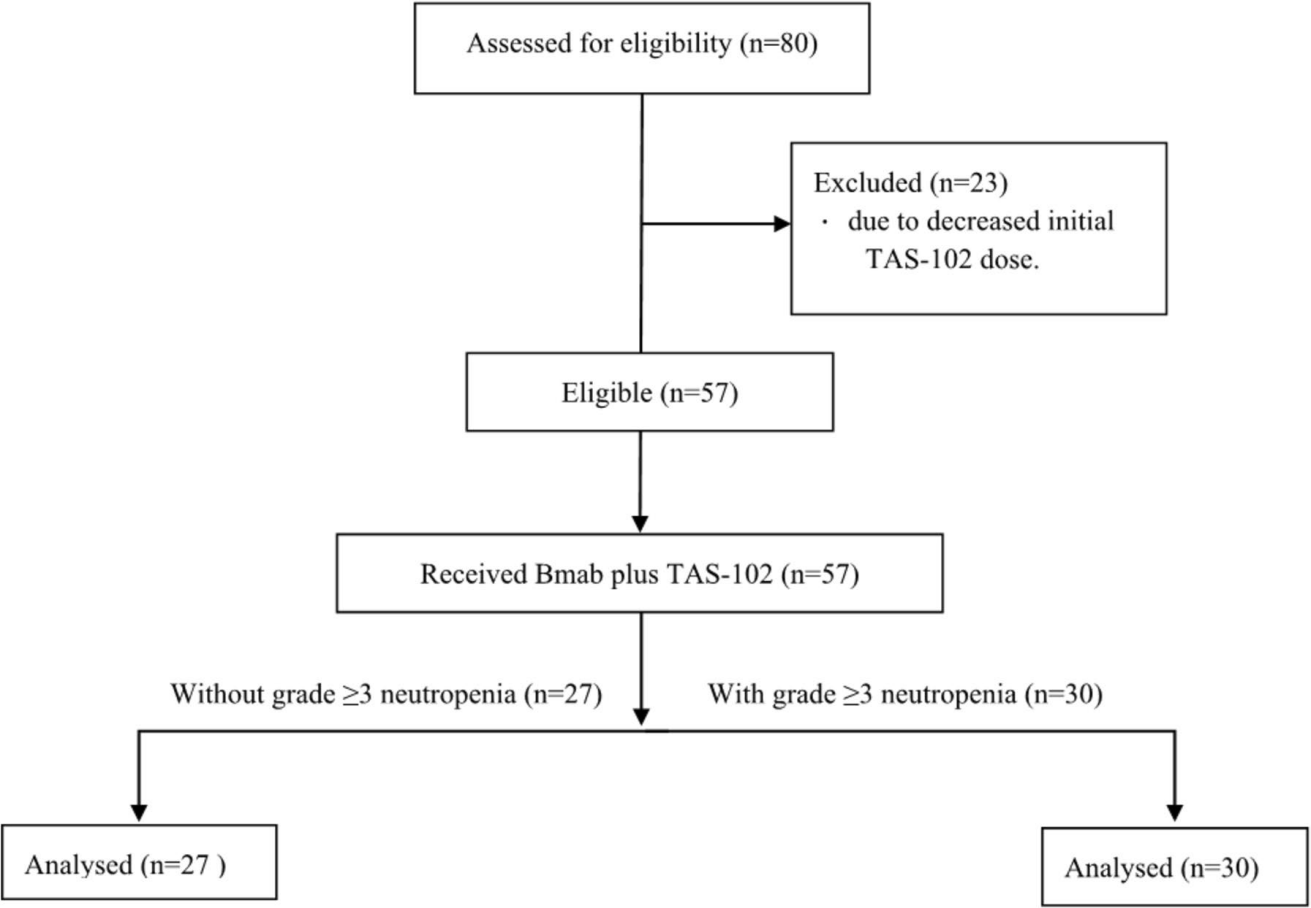
Consolidated Standards of Reporting Trials (CONSORT) Flow Diagram. Abbreviation: Bmab, bevacizumab

An eligible cohort of 80 patients with metastatic colorectal cancer who underwent TAS-102 treatment was identified and assessed for inclusion (Fig. 1). Subsequently, 23 subjects were excluded due to a reduced initial dose of TAS-102. The study cohort was divided based on the onset of severe neutropenia during the follow-up period into 30 patients with severe neutropenia and 27 patients without severe neutropenia. All 57 subjects who met the eligibility criteria were included in the final analysis. Baseline demographics are shown in Table 1. Except for one patient with no RAS, BRAF, or MSI data, there were no missing baseline data.

**Table 1:** Baseline characteristics of patients with and without grade ≥ 3 neutropenia

**Variable**	**Overall**, *N* = 57^a^	**With grade ≥ 3 neutropenia**, *N* = 301	**Without grade ≥ 3 neutropenia**, *N* = 27^a^
**Age, year**	67 (55, 74)	65 (55, 75)	68 (52, 74)
**Sex**			
Male	41 (71.9%)	21 (70.0%)	20 (74.1%)
Female	16 (28.1%)	9 (30.0%)	7 (25.9%)
**Albumin, mg/dL**	3.9 (3.5, 4.1)	3.8 (3.5, 4.0)	3.9 (3.6, 4.1)
**Aspartate aminotransferase, IU/L**	25.0 (21.0, 35.0)	24.0 (19.2, 31.2)	27.0 (21.5, 38.5)
**Alanine aminotransferase, IU/L**	17.0 (12.0, 30.0)	16.5 (12.0, 25.0)	18.0 (13.0, 46.5)
**Total bilirubin, mg/dL**	0.6 (0.5, 0.8)	0.7 (0.6, 0.9)	0.5 (0.4, 0.8)
**Creatinine clearance**^b^	79.9 (67.5, 114.3)	77.5 (61.1, 90.3)	94.4 (70.6, 128.6)
**CRP, mg/dL**	0.5 (0.2, 1.8)	0.4 (0.2, 0.8)	1.1 (0.2, 4.9)
**mGPS**			
**0**	28 (49.1%)	17 (56.7%)	11 (40.7%)
**1**	21 (36.8%)	9 (30.0%)	12 (44.4%)
**2**	8 (14.0%)	4 (13.3%)	4 (14.8%)
**Metastases**	51 (89.5%)	29 (96.7%)	22 (81.5%)
**RAS mutation**	26 (46.4%)	14 (46.7%)	12 (46.2%)
**BRAF mutation**	2 (3.6%)	0 (0.0%)	2 (7.7%)
**MSI high**	1 (1.8%)	0 (0.0%)	1 (3.8%)
**Previously treated with regorafenib**	7 (12.3%)	6 (20.0%)	1 (3.7%)

*Abbreviations*: *mGPS* Modified Glasgow prognostic score

^a^n (%); Median (IQR)

^b^The Cockroft-Gault equation was used to estimate creatinine clearance based on serum creatinine and patient characteristics

### Survival analysis and incidence of grade ≥ 3 neutropenia in the entire cohort

In all study subjects, the median duration of follow-up was 305 days, with an interquartile range of 217–432. Median OS was 14.2 months (95% CI [13.4 – 18.6]) and median PFS was 6.8 months (95% CI [5.2 – 10.2]).

The onset of severe neutropenia during the follow-up period was 52.6% (30/57). Among the 30 patients, the median onset time of severe neutropenia from the initiation of Bmab + TAS-102 was 56 days, with an interquartile range of 28 to 70.

### Comparison of the efficacy of the combination TAS-102 and Bmab in patients with and without severe neutropenia

**Fig. 2:**
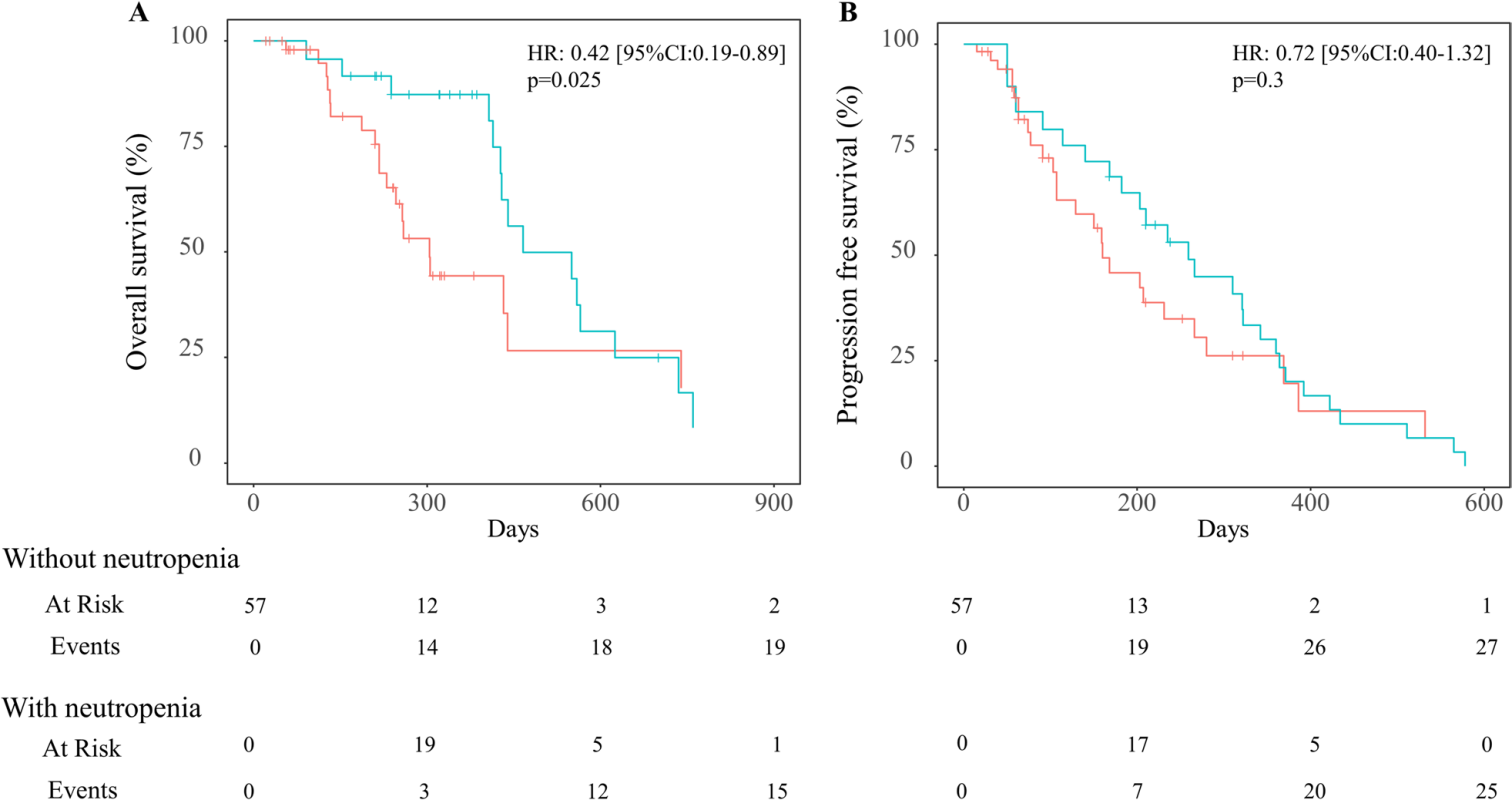
Simon and Makuch’s modified Kaplan–Meier curves for overall survival (**A**) and progression-free survival (**B**) in colorectal cancer patients who received a combination of trifluridine-tipiracil and bevacizumab. One curve (green line) shows patients who experienced grade ≥ 3 neutropenia during the treatment period (30 patients [52.6%] with grade ≥ 3 neutropenia), and the other curve (red line) shows those who did not (27 patients [47.4%] without grade ≥ 3 neutropenia). Abbreviations: Bmab + TAS-102, trifluridine-tipiracil in combination with bevacizumab. CI, confidence interval. HR, hazard ratio. mGPS, modified Glasgow prognostic score. NA, indicates calculation not possible. OS, overall survival. PFS, progression-free survival

To prevent immortal-time bias in assessment of the association between neutropenia and survival, we implemented both a time-varying Cox regression model and landmark analysis. Median survival was determined using Simon and Makuch’s modified Kaplan–Meier survival curves. Results showed that median OS was 15.3 months [95% CI: 14.1–NA] for patients with severe neutropenia and 10.0 months [95% CI: 8.1–NA] for those without severe neutropenia. Time-varying Cox regression showed that severe neutropenia was significantly associated with longer survival after adjusting for age and mGPS (HR: 0.42; [95% CI: 0.19–0.89], *p* = 0.025) (Fig. 2A). Median PFS was 8.5 months [95% CI: 5.5–12.0] and 5.3 months [95% CI: 3.5–9.2] for patients with and without severe neutropenia, respectively. Although not significant, patients with severe neutropenia tended to have longer PFS (HR: 0.72; [95% CI: 0.40–1.32], *p* = 0.3) (Fig. 2B).

Next, 30-, 60-, 90-, and 120-day landmark analyses were performed. Median survival time between groups with and without neutropenia at the 30-, 60-, 90-, and 120-day landmarks was 19.6 [13.1-NA] and 13.1 [7.5–17.4], 16.1 [12.1-NA] and 12.2 [8.0–16.6], 15.1 [11.1-NA] and 10.7 [5.5–21.3], and 11.4 [10.2-NA] and 9.7 [4.5–20.4], respectively. Cox proportional hazards analysis showed HRs for the association between neutropenia and OS at the 30-, 60-, 90-, and 120-day landmarks of 0.30 [0.10–0.90], 0.65 [0.30–1.42], 0.39 [0.17–0.90], and 0.41 [0.18–0.95], respectively (Table 2).

**Table 2:** Survival analysis at 30, 60, 90, and 120-day landmarks

**Landmark time point, days**	**Number of patients** ^a^		**Median survival time after landmark period, months** ^b^		**Hazard ratio** ^c^ **(95% CI)**
	With grade ≥ 3 neutropenia	Without grade ≥ 3 neutropenia	With grade ≥ 3 neutropenia	Without grade ≥ 3 neutropenia	
30	9 (15.8)	48 (84.2)	19.6 (13.1–NA)	13.1 (7.5–17.4)	0.30 (0.10–0.90)
60	17 (30.4)	39 (69.6)	16.1 (12.1–NA)	12.2 (8.0–16.6)	0.65 (0.30–1.42)
90	23 (41.1)	33 (58.9)	15.1 (11.1–NA)	10.7 (5.5–21.3)	0.39 (0.17–0.90)
120	24 (44.4)	30 (55.6)	11.4 (10.2–NA)	9.7 (4.5–20.4)	0.41 (0.18–0.95)

*Abbreviations*: *CI* Confidence interval, *mGPS* Modified Glasgow prognostic score, *NA* Calculation not possible

^a^n (%)

^b^median (confidence interval)

^c^Hazard ratio and 95% confidence interval was estimated by Cox proportional hazards regression adjusted age and mGPS

### Comparison of overall response rate, relative dose intensity, and usage of regorafenib following TAS-102 with bevacizumab between patients with and without severe neutropenia

Table 3 shows the overall response rate, disease control rate, relative dose intensity, and use of regorafenib following treatment with Bmab + TAS-102 in patients with and without severe neutropenia. Although not significant, patients with severe neutropenia tended to have higher disease control rates (90.0% vs. 70.4%, *p* = 0.093). Notably, the relative dose intensity of TAS-102 was significantly lower in patients with severe neutropenia (0.73 vs 0.84, *p* = 0.014). The proportion of patients who received regorafenib following Bmab + TAS-102 in the two groups was comparable (33.3% vs. 33.3%, *p* > 0.9). The occurrences of other adverse events among patients with and without severe neutropenia are shown in Supplemental Table 1.

**Table 3:** Comparison of overall response rate, relative dose intensity, and regorafenib use post-treatment

**Variable**	**Overall**, *N* = 57^a^	**With grade ≥ 3 neutropenia**, *N* = 30^a^	**Without grade ≥ 3 neutropenia**, *N* = 27^a^	***p*-value**^2^
**Overall response rate**				0.093
Partial response	1 (1.8%)	1 (3.3%)	0 (0.0%)	
Stable disease	45 (78.9%)	26 (86.7%)	19 (70.4%)	
Progressive disease	11 (19.3%)	3 (10.0%)	8 (29.6%)	
**Disease control rate**	46 (80.7%)	27 (90.0%)	19 (70.4%)	0.093
**Relative dose intensity**				
Trifluridine-tipiracil	0.76 (0.69, 0.89)	0.73 (0.62, 0.82)	0.84 (0.75, 0.89)	0.014
Bevacizumab	0.75 (0.57, 0.88)	0.75 (0.60, 0.86)	0.75 (0.56, 0.89)	0.7
**Regorafenib use in post-treatment**	19 (33.3%)	10 (33.3%)	9 (33.3%)	> 0.9

^a^n (%); Median (IQR)

^2^Fisher’s exact test; Wilcoxon rank sum test

## Discussion

The objective of this study was to examine the impact of severe neutropenia on the efficacy of treatment with Bmab + TAS-102 while addressing the problem of immortal time bias. To overcome this challenge, we applied two strategies: a time-varying Cox regression model and landmark analysis [18]. The results of these two methods were consistent and indicated that grade ≥ 3 neutropenia onset was significantly associated with longer survival.

Two other studies have examined the relationship between neutropenia during Bmab + TAS-102 therapy and OS. Their results warrant caution, however, as they either did not adjust for confounding variables [8] or for the time-varying nature of neutropenia [6]. Our study is distinctive in that it addressed immortal time bias, and controlled for major confounding factors including age and mGPS by time-varying Cox proportional hazards regression. In addition, our 30-, 60-, 90-, and 120-day landmark analyses showed that neutropenia during the early period after the first cycle was associated with prognosis. These results provide novel evidence in support of these previous findings of an association between prognosis and neutropenia during the first cycle of TAS-102 monotherapy [7] and in combination with Bmab [8]. In this regard, our study addresses the limitations of prior research and contributes to a more robust evidence base.

In our study, patients with severe neutropenia exhibited significantly lower relative dose intensity for TAS-102 due to dose reductions in response to severe neutropenia. Nevertheless, disease control rates in these patients were higher, suggesting that reducing the dose in response to grade ≥ 3 neutropenia could be acceptable from a risk–benefit perspective. Conversely, for patients who do not experience neutropenia, the standard dose of TAS-102 may not be sufficient to induce myelotoxicity, and an increase in TAS-102 dosage as a potential strategy to improve treatment outcomes may be considered, as discussed by Kasi et al. [7, 19]. The relationship between neutropenia and patient outcomes may be explained by neutropenia's potential role as an indirect measure for the optimal dosage of Bmab + TAS-102. This marker could reflect various factors such as drug exposure, dose density, and metabolic activity, as indicated in prior studies on other colorectal cancer treatments [20, 21]. Specific gene variations related to the metabolism of trifluridine and the excretion of tipiracil have been shown to affect the effectiveness and toxicity of TAS-102 in patients with metastatic colorectal cancer [22, 23]. Interestingly, a higher incidence of neutropenia has been correlated with both a higher area under the curve for trifluridine plasma concentration and longer OS [24]. Consequently, use of body surface area alone to determine TAS-102 dosage may not be adequate, as it fails to consider individual differences in drug metabolism.

As one result of our study, we found that severe neutropenia exhibited a favorable yet statistically non-significant impact on PFS, with a more pronounced effect on OS. These findings lead us to hypothesize that Bmab + TAS-102 therapy, coupled with the onset of neutropenia, may modulate the tumor microenvironment. Recent research indicates that TAS-102 exerts its antitumor immune effect primarily by directly eliminating Tumor-Associated Macrophage 2 (TAM2) [25], which promotes tumor growth through mechanisms such as angiogenesis and immunosuppression [26, 27]. VEGF inhibitors, such as Bmab, enhance antitumor responses by inhibiting the infiltration of tumor-promoting immune cells, including TAM2 [28]. Neutrophils are also known to contribute to tumor growth by promoting angiogenesis and suppressing antitumor immune responses [29–31]. Therefore, neutropenia induced by Bmab + TAS-102 may alter the tumor microenvironment and potentially influence tumor evolution following treatment [32].

Our study has several limitations. First, it was conducted under a retrospective design at a single center. Second, due to the small sample size, the number of variables included in the multivariable analysis was restricted to avoid overfitting. As a result, we could not adjust for additional factors, such as comorbidities. Third, given that the OS and PFS in our study were longer than those reported in a previous phase III trial (median OS: 9.4 months, median PFS: 4.6 months) [4], it is possible the target population in this study had a relatively favorable overall health status that would be considered acceptable for Bmab combination therapy. Fourth, the mechanism underlying the relationship between myelosuppression and increased antitumor activity remains unresolved. In particular, it is yet to be established whether myelosuppression enhances antitumor activity by suppressing tumor-promoting cells, such as TAM, or whether severe neutropenia develops as a consequence of administering an optimal dose of TAS-102. Further investigation in larger cohorts is necessary to validate the outcomes of this study, clarify the molecular mechanisms underlying the impact of Bmab + TAS-102-induced severe neutropenia on prognosis, and evaluate the potential benefit of modifying subsequent TAS-102 doses in response to hematological toxicity during treatment.

## Conclusion

Our study showed a significant association between the occurrence of severe neutropenia during Bmab + TAS-102 and long-term survival, utilizing an approach that addresses immortality time bias.

### Supplementary Information

**Additional file 1: Table S1.** The occurrences of adverse events among patients with and without grade ≥3 neutropenia.

## Data Availability

The data that support the findings of this study are available from the study groups, but restrictions apply to the availability of these data, which were used under license for the current study; therefore, the data are not publicly available. However, data are available from the corresponding authors upon reasonable request and with permission from the study groups.

## Abbreviations

Bmab + TAS-102: Trifluridine-tipiracil in combination with bevacizumab
CI: Confidence interval
HR: Hazard ratio
mGPS: Modified Glasgow prognostic score
NA: Calculation not possible
OS: Overall survival
PFS: Progression-free survival
TAM2: Tumor-associated macrophage 2
