# Supplementary material for: Prognostic impact of severe neutropenia in colorectal cancer patients treated with TAS-102 and bevacizumab, addressing immortal-time bias
Source: BMC Cancer. 2023 Nov 8;23:1078. doi: 10.1186/s12885-023-11618-3 (PMC10634158; doi:10.1186/s12885-023-11618-3)
Supplement: Supplementary file 1 — Additional file 1: Table S1. The occurrences of adverse events among patients with and without grade ≥3 neutropenia. [file 12885_2023_11618_MOESM1_ESM.docx]

Table S1. The occurrences of adverse events among patients with and without grade ≥3 neutropenia.

| **Adverse events (grade ≥2)** | **Overall, N = 57^1^** | **Without grade ≥3 neutropenia, N = 27^1^** | **With grade ≥3 neutropenia, N = 30^1^** |
| --- | --- | --- | --- |
| **Nausea** | 23 (40.4%) | 14 (51.9%) | 9 (30.0%) |
| **Diarrhea** | 13 (22.8%) | 3 (11.1%) | 10 (33.3%) |
| **Fatigue** | 18 (31.6%) | 7 (25.9%) | 11 (36.7%) |
| **Hypertension** | 16 (28.1%) | 3 (11.1%) | 13 (43.3%) |
| **Mucositis** | 6 (10.5%) | 2 (7.4%) | 4 (13.3%) |
| **Dysgeusia** | 3 (5.3%) | 2 (7.4%) | 1 (3.3%) |

^1^n (%)
